# Analyses of Acceptability Judgments Made Toward the Use of Nanocarrier-Based Targeted Drug Delivery: Interviews with Researchers and Research Trainees in the Field of New Technologies

**DOI:** 10.1007/s11569-015-0241-2

**Published:** 2015-10-29

**Authors:** Vanessa Chenel, Patrick Boissy, Jean-Pierre Cloarec, Johane Patenaude

**Affiliations:** Interdisciplinary Institute for Technological Innovation (3IT), Université de Sherbrooke, Sherbrooke, QC Canada; Department of Surgery, Faculty of Medicine and Health Sciences, Université de Sherbrooke, 3001-12th Avenue North, Sherbrooke, QC J1H 5N4 Canada; Laboratoire Nanotechnologies et Nanosystèmes (LN2), Centre National de la Recherche Scientifique (CNRS), Université de Sherbrooke, Sherbrooke, QC Canada; Institut des Nanotechnologies de Lyon (INL), site École Centrale de Lyon, Université de Lyon, Lyon, France

**Keywords:** Acceptance, Impact perception, Expert perception, Nanomedicine, Qualitative research, E^3^LS

## Abstract

The assessment of nanotechnology applications such as nanocarrier-based targeted drug delivery (TDD) has historically been based mostly on toxicological and safety aspects. The use of nanocarriers for TDD, a leading-edge nanomedical application, has received little study from the angle of experts’ perceptions and acceptability, which may be reflected in how TDD applications are developed. In recent years, numerous authors have maintained that TDD assessment should also take into account impacts on ethical, environmental, economic, legal, and social (E^3^LS) issues in order to lead to socially responsible innovation. Semi-structured interviews (*n* = 22) were conducted with French and Canadian researchers and research trainees with diverse disciplinary backgrounds and involved in research related to emerging technologies. The interviews focussed on scenarios presenting two types of TDD nanocarriers (carbon, synthetic DNA) in two contexts of use (lung cancer, seasonal flu). Content and inductive analyses of interviews showed how facets of perceived impacts such as health, environment, social cohabitation, economy, life and death, representations of the human being and nature, and technoscience were weighed in acceptability judgments. The analyses also revealed that contextual factors related to device (nature of the treatment), to use (gravity of the disease), and to user (culture) influenced the weighting assigned to perceived impacts and thus contributed to variability in interviewees’ judgments of acceptability. Giving consideration to researchers’ perspective could accompany first steps of implementation and development of nanomedicine by producing a first, but wide, picture of the acceptability of nanocarrier-based TDD.

## Introduction

Nanomedicine, defined as the application of nanotechnologies (NT) to health care and medical research [1], is likely to experience significant development in the near future [2]. The technical capabilities of NT currently being reported are extremely promising, and expectations for nanomedicine (NM) run high. However, the potential consequences of the development and use of nanomedical technologies are likely to extend beyond the safety and toxicological issues traditionally taken into account by regulatory and assessment bodies, such as the FDA, and to affect society in more wide-ranging ways [3]. NM remains a novel concept and uncertainty still surrounds both the technical and the societal impacts of the deployment of NM, of which targeted drug delivery (TDD) appears to be the most promising [4]. As such, the NM development process should include the study of the ethical, legal, and social implication (ELSI), as well as environmental health and safety issues (EHS) and other scientific, economic, and political concerns [5]. In order to exceed the limitations of this segmented ELSI/EHS approach [6], where ELSI and EHS issues are normally analyzed independently, this paper presents an integrative approach where ethical, environmental, economic, legal, and social (E^3^LS) issues are considered as a whole. Consequently, this reorientation calls upon an interdisciplinary joint effort on issues related to emerging technologies, such as NM, from the very first stages of development. Moreover, if integrated into the technological development process, an E^3^LS approach would allow the elaboration of more value-driven applications, exceeding the traditional limitation of cost-benefit evaluations.

A more thorough examination of the range of possible impacts of the development and use of TDD nanocarriers could lead to a better understanding of which facets people tend to prioritize in connection with this application. Thus, possible divergences between current avenues of scientific exploration and concerns in society at large can be anticipated. *Acceptability* is defined as a value judgment reflecting the weighting assigned to the various factors that are taken into consideration in reaching a decision about the personal use of a given technology or that contribute to one’s perspective on what is desirable for society [7]. Beyond revealing perceived impacts, the analysis of acceptability by experts in the field of emerging technologies, who are also citizens and potential final users, could determine a spectrum of factors taken into account and weighed in reaching acceptability judgments about NM applications. Such a spectrum could then help to identify areas affecting acceptability and guide innovators to make responsible, informed choices and create a forum for social dialogue on questions emerging from technological development.

The perception and acceptability of NM applications has not been investigated in depth to date. In a study that presented 20 NT-based applications (including TDD nanocarriers), factors influencing the perception of nanotechnology hazards were examined in both laypeople and experts [8]. Siegrist et al. investigated the impact of perceived risks and benefits in relation to other factors such as social trust and technology fears. Their factor analysis allowed the classification of applications according to the characteristics dread risk and distrust. Their study did not, however, aim to describe the perception of the impacts directly related to the applications presented.

A more recent study that presented eight applications, some of which were NM-related, revealed the influence of the nature of risks and benefits presented on the acceptability of the different applications [9]. Again, the research objectives did not aim at portraying perceived impacts for each application. Nerlich et al. showed that among young people, the frequency of administration of a treatment is likely to influence attitudes more heavily than the treatment’s “nano” nature [10]. While NM elicited less enthusiasm than expected, the adverse effects of conventional treatment were deemed to be important in long-term use, and the positive effects of a nanotreatment were deemed to override adverse effects in long-term use. On the other hand, Bottini et al. showed that NM and its applications elicit public optimism similar to that felt toward NT [11]. This optimism, however, is accompanied by concerns regarding health, environmental, and social aspects. A study of perceptions of NT and NM conducted on statements posted to a popular social media site revealed that a large number of users perceived NM’s potential economic impacts, and close to 50 % of users considered theranostics (the use of nanosystems, capable of diagnosis, drug delivery, and monitoring of therapeutic response [12]) to be one of the most promising nanomedical application [13]. Finally, the perceptions of practitioners in the field of NM were studied from the perspective of ethical aspects of their clinical practice. The work of Silva Costa et al. suggests that these practitioners are interested in reflecting on ethical issues in their work but overall do not consider ethical issues associated with NM to be new and solely associated to NM [14]. Overall, the investigation of risks and benefits has mostly focussed on the establishment of general relationships between impacts and the apprehension and awareness of respondents toward the applications presented. The qualitative nature of some issues and the implications related to perceived impacts remain unexplored. Those impacts are not external to the emerging technologies. Because they are characteristic of how those technologies are developed and used, it appears important to identify, describe, and understand them (regardless of whether they are known, probable, hypothetical, or theoretical), which could constitute a first step toward responsible innovation [7].

Acceptability judgment in relation to technological applications has also been scarcely studied, even less when we consider nanotechnological applications. In 1978, Fischhoff et al. recognized the significance of the risk-acceptance judgment process based on an assessment of certain characteristics of the technology or threat being studied (i.e., the risk’s dread nature, voluntariness of exposure, and the risk’s controllability) [15]. Rooted in a psychometric paradigm that emphasizes the influence of individual and social characteristics on perceived risks, this approach aims to predict acceptance (defined as a statement of fact regarding the use of a technology) of users rather than to understand the acceptability judgment of respondents (what would be defined as the weighing of perceived impacts). In more recent work, Senjen and Hansen [16] argue for the importance of taking metaphysical risks (i.e., modification of the representation of the human being) into account when a judgment must be formed about risks. They suggest that many value judgments may come into play and differ among themselves by virtue of the factors that enter into play. They also suggest that both public and expert perceptions could account for a broader range of values in assessing technologies. This perspective is illustrated in Bjornstad and Wolfe [17], who demonstrate that a medical technology that represents a major advance over existing products could, on one hand, offer unprecedented solutions for certain health problems while, on the other hand, come into conflict with individual and social values. According to Giacomini et al. [18], in a pluralist society, a clarification of the individual and societal values that underlie a technology’s acceptability could offer a way to answer complex questions about how to allot limited health care resources and how to steer innovations in the health field. Going beyond the question of the nature of the factors considered in forming an acceptability judgment, work has been done on variability in acceptability judgments. It has been found that the judgment process among experts who assess risk as the expected value of the negative outcomes of a decision may vary between individuals, across contexts, and over time, which suggests the existence of modulating factors that influence an assessor’s value judgments [19]. Context of use and disciplinary culture have also been shown to be factors likely to influence acceptability judgments about the use of nanocarriers for TDD [20, 21]. Still, other research results support the view that public perceptions of NT shift according to the application being assessed and the assessor [9]. Despite this literature on acceptability, little is known about what impacts are taken into account in potential users’ acceptability judgment regarding NM and about how those judgments are formed; further, even though some theoretical literature exists on that topic, few studies have considered the practical dimensions of the research.

In light of this review of the literature, a gap exists regarding both the set of impacts actually perceived by potential users of nanomedical applications and the way users weigh those impacts in arriving at an acceptability judgment. Given the recognized fact that no impact can be perceived and prioritized in an equal manner either in all the fields of application for NT [22] or by all users, it is appropriate to expand the study of impact perception around acceptability of NM, seeing as these bear on each application, while paying special attention to the individual profiles of assessors and to the context of use. The perspective of researchers on NM has not been the study of numerous studies [14, 23] and no work has been done to understand how they formulate their acceptability judgments and what they do take into account in arriving in such judgments. Given the social innovations context within which NM is drafted, the assessment of acceptability by researchers in the field of emerging technologies may help in aligning the implementation and development of technologies such as nanotechnologies with societal preferences [23].

This paper presents qualitative results for the third component of a mixed-methods study with sequential data triangulation that included the administration of a web-based questionnaire and in-depth semi-structured interviews. The sample was composed of people conducting research, as academic professionals or as research trainees. The design of this mixed-methods study was built around the analysis of scenarios that presented a nanomedical application, namely, nanocarrier-based TDD. Scenarios described two kinds of envisioned TDD nanocarriers (carbon-based, synthetic DNA-based) in two clinical contexts of use (treatment of lung cancer, treatment of seasonal flu). The envisioned nanocarriers have been inspired by existing and current research and selected regarding their potential to affect acceptability judgment, based on the possible values behind their composition (carbon, synthetic DNA). Lung cancer and seasonal flu have been chosen as case studies because they represent generally known and common diseases affecting the same body region, yet at a different level (pulmonary system). Using an E^3^LS theoretical framework for the analysis of the impacts and acceptability of technological applications [7], the first component of the study presented impact perceptions, acceptance, and acceptability judgments of researchers in relation to the two kinds of nanocarriers presented in the two contexts of use [20]. The second component examined the impact of disciplinary culture on the impact perceptions, acceptance, and acceptability judgments of researchers in relation to the same applications presented under the same context of use [21]. The main objective of this third component was to document in a more detailed fashion the impacts and impact facets perceived and weighed by researchers in the field of new technologies when arriving at an acceptability judgment about envisioned applications of TDD. The collateral objective was to identify contextual factor that may have modulated their acceptability judgment.

## Methods

### Sample

The main sample of the study was composed of respondents conducting research related to emerging technologies and from several cultural and disciplinary backgrounds. Regarding the spectrum of possible E^3^LS impacts related to the development and the use of NM applications, respondents were targeted from across natural sciences and engineering (NSE), and social sciences and humanities (SSH). NSE researchers were targeted based on their work in connection with the development of nanotechnological applications; SSH researchers were targeted based on their work in connection with the study of emerging technologies. Also, the increasing number of international collaborations oriented the choice of a culturally diverse sample; respondents were targeted from across Europe and Canada. Since the questionnaire was elaborated in French, only Francophone researchers in Canada and Europe were targeted. In the quantitative phase of the study, email invitations were sent to researchers and research trainees in the field of new technologies. (Hereafter, the word *researcher* will cover all respondents, including research trainees.) A researcher was defined as an author of publications on themes related to new technologies; a scientist who participates in conferences; or a member of a research group, lab, or network (such as NE^3^LS Network on Nanotechnology [24], Canada, and PACTE - Social Science Research Laboratory [25], France). The researchers (*n* = 1320) were asked to fill out the web-based questionnaire. Of this number, 214 filled out the questionnaire meeting the minimum completion quality outcome defined a priori.

A separate section at the end of the questionnaire informed respondents of the possibility of contributing to the study’s second phase and allowed interested respondents to provide contact details. The final number of participants for the interviews was based on data saturation. Two criteria were applied in choosing participants for the qualitative phase: equal representation of the two social cultures (European, Canadian), and equal representation of the two broad disciplinary spheres (NSE, SSH). To ensure diversity in disciplinary backgrounds, researchers were recruited from anthropology, applied ethics, bioethics, biology, chemistry, electric engineering, informatics, medicine and nanomedicine, microbiology, neuroethics, philosophy, physics, and sociology of sciences (see Table 1). The final subsample for the qualitative phase (*n* = 22) comprised 11 French NSE (*n* = 6) and SSH (*n* = 5) researchers and 11 Canadian NSE (*n* = 5) and SSH (*n* = 6) researchers. Of the 22 interviewees, 16 were academic researchers and 6 were research trainees. Ten participants were between 25 and 34 years old, two were between 35 and 44 years old, six were between 45 and 54 years old, three were between 55 and 64 years old, and one was 65 or older. Male respondents accounted for 86 % of the sample. The two phases of the study were approved by a research ethics board of the Centre Hospitalier Universitaire de Sherbrooke (CHUS), and participants provided their consent to participate.

**Table 1:** Brief profile of researchers and research trainees

SI. no.	Disciplinary culture	Disciplinary background	Social culture
QNSEF03	NSE	Biology – Nanomedicine	France
QNSEF02	NSE	Chemistry – Nanomaterials	France
QNSEF01	NSE	Chemistry – Nanosensors	France
QNSEC03	NSE	Chemistry engineering – Nanotechnology	Canada
QNSEC01	NSE	Electric engineering – Nanotechnology	Canada
QNSEC05	NSE	Electric engineering – Nanotechnology	Canada
QNSEF05	NSE	Informatics – Biotechnology	France
QNSEF04	NSE	Medicine – Radiation oncology	France
QNSEC02	NSE	Microbiology – Nanosensors	Canada
QNSEF06	NSE	Nanomedicine – Biomimicry	France
QNSEC04	NSE	Process chemistry	Canada
QSSHC04	SSH	Applied ethics – Neuroethics	Canada
QSSHC02	SSH	Bioethics – Clinical research	Canada
QSSHC01	SSH	Bioethics – Epigenetics	Canada
QSSHC05	SSH	Ethics – Anthropology	Canada
QSSHC06	SSH	Ethics – Technological innovation	Canada
QSSHF03	SSH	Human factors and ergonomics	France
QSSHF02	SSH	Philosophy – Applied Ethics	France
QSSHF05	SSH	Philosophy – Applied Ethics	France
QSSHC03	SSH	Philosophy – Applied Ethics	Canada
QSSHF04	SSH	Physics – Ethics of nanotechnology	France
QSSHF01	SSH	Sociology of sciences	France

*NSE* Natural Sciences and Engineering, *SSH* Social Sciences and Humanities, *SI. no.* subject identification number

### Data Collection and Analyses

All the interviews were conducted by the first author and lasted on average 1 h. Most of the interviews were conducted in person in a location that suited the participant. When in-person interviewing was impossible, teleconferencing was used (*n* = 7). A consent form was read and signed before beginning the interview. An interview guide with open-ended questions was devised following a preliminary analysis of the results of the web-based questionnaire. This guide was followed in all the interviews. Probes allowed for exploring the various facets of the acceptability judgment. Notes on emergent themes and contextual factors were taken throughout the interviews. The audiotaped interviews were professionally transcribed verbatim. Once the transcripts had been reviewed for accuracy by playing back the audio recordings, a process of data immersion through the attentive reading of the transcripts yielded a solid acquaintance with each interview’s themes and content. Throughout the transcription and preparation process, data was anonymized in order to remove all information that might result in the identification of participants.

Two approaches were used sequentially to analyze the data. The data that related to the identification of the impacts involved in acceptability judgments were first subjected to thematic content analysis in order to arrive at an objective and systematic description of the interviewees’ discourse [26] by identifying, inventorying, and classifying the content components [27]. A mixed coding method based on the existence of general themes derived from the governing theoretical framework [7] allowed for the emergence of codes related to the theme of acceptability. Next, in view of the study’s exploratory nature and in the absence of a theoretical framework regarding the contextual factors that modulate the acceptability judgment [28], a general inductive approach to data analysis was preferred for processing the raw qualitative data, thus satisfying the study’s second objective [29]. The primary purpose of the general inductive approach is to allow research findings to emerge from the frequent, dominant, or significant themes inherent in raw data, without the restraints imposed by structured methodologies. The outcome of such analysis is the identification of the main themes related to the research objectives [29]. Open coding was preferred for this analysis. Most of the codes emerged from the compilation of interviews and their associated memos. A reading and systematic coding of the transcripts resulted in the emergence of the major themes. No new themes emerged as the end of the study drew near suggesting that the major contextual factors likely to modulate acceptability judgments about the use of TDD nanocarriers in the scenarios presented had all been identified. All analyses were performed by the first author using the qualitative data analysis software Dedoose v4.12 (SocioCultural Research Consultants, UCLA, CA).

## Results

### The Issues and Impacts Considered in Forming Acceptability Judgments

A large number of both positive and negative impacts on seven issues were considered by the interviewees to arrive at their acceptability judgments. These impacts emerged following a first encounter with the transcripts; they were grouped under issues identified and named based on pre-existing categories emerging from the theoretical framework that governed the study. In addition to health, environmental, and social issues initially raised in the web-based questionnaire, the interpretation of the interview contents yielded the identification of impacts related to the economy, life and death, representations of the human being and nature, and technoscience. The identification of these seven foundational issues in the acceptability judgments of respondents allowed for a first-level classification and the ensuing initial interview coding, from which we derived a list of analysis units grouped by issue. A progressive and iterative process of analyzing the units of analysis for each category of issue resulted in the extraction of what was essential to each category. In this way, a set of impacts specific to each issue was identified. A second coding process was conducted based on this analysis, and the identification of more precise meaning units related to each impact allowed for the description of the impacts invoked for all seven issues. In continuity with the coding process, a final iteration of the analytic process resulted in the fine-tuning of each of the impacts and in the presentation of the impact facets. This process of thematic analysis laid out, in a systematic manner, a range of impacts and of impact facets as taken into account and prioritized by the researchers in forming their acceptability judgments about the scenarios presented. Table 2 presents all impacts and impact facets that go into the composition of acceptability judgments.

**Table 2:** Impacts and impact facets weighed in acceptability judgments about the two types of nanocarriers as used in the two clinical situations

Issue	Impact	Facets
Health	Desirable effects	*Recovery*
		*Mitigation of symptoms*
		*Less significant adverse effects*
	Well-being	*Degree of comfort associated with treatment’s administration*
		*Enhanced quality of life*
		*Greater compliance with treatment*
	Undesirable effects	*Bioaccumulation*
		*Systemic toxicity*
		*Genetic toxicity*
	Body’s homeostasis	*Disturbance of established order*
		*Effect on immune system’s autonomy*
	Workers’ health	*Individuals in production chain*
		*Individuals in treatment chain*
Environment	Pollution	*At time of production*
		*At time of elimination from patient’s body*
	Sustainable development	*–*
Social cohabitation	Access/inequality	*Developing country*
		*Differences between social classes*
	Treatment’s social burden	*–*
	Social impact of the disease	*–*
	Possibility of choosing the treatment	*–*
	Sick people’s productivity	*Individual with key role in society*
		*Unnecessary return to work*
Economy	Development costs	–
	Treatment costs	*Cost to the individual at time of purchase*
		*Reimbursement by insurance plan*
	Economic attractions	*For pharmaceutical companies*
		*For national economy*
Life–death	Life expectancy	*Increased in the short term*
		*Reduced in the long term*
	Treatment for a fatal disease	*–*
	Death in the wake of the treatment	*–*
Representations of human being and nature	Identity/self	*Alien substance in human body*
		*Artificial substance*
	Body modification	*–*
	Definitions of health/sickness	*–*
Technoscience	Advancement of science	*For its own sake*
		*So that other advances may follow*
	Questioning of treatment	*–*

#### Health

Health impacts related to desirable and undesirable effects for the treated person, to the treated person’s well-being, to the treated person’s bodily homeostasis, and to the health of the workers involved in producing and administering the treatment were all considered by the interviewees in arriving at their acceptability judgments. Each of these impacts is defined by a range of facets. The possibility of recovery, the mitigation of symptoms, and the occurrence of less significant adverse effects are facets that were considered in connection with desirable effects and that could count toward a positive judgment about the applications in the scenarios. The degree of comfort associated with the method of administration, enhanced quality of life, and greater compliance with treatment are factors associated with the respondent’s well-being. These factors, while not associated with the therapeutic response to treatment, can have a positive effect on a sick person’s health. Respondents who expressed an acceptability judgment in favor of the use of the treatments presented placed emphasis on these facets. On the other hand, a counterweight was provided by undesirable effects in the form of the risk of the bioaccumulation of nanocarriers in the human body and the adverse effects resulting from systemic or genetic toxicity. Respondents who viewed these facets as outweighing the desirable effects appeared to have judged nanocarriers to be less acceptable. For some respondents, the impact of the treatment on internal homeostasis through its disturbance of the established order and through the possibility of an effect on the immune system’s autonomy also entered into the acceptability judgment, especially for the ones who disagreed with the use of nanocarriers for TDD to treat seasonal flu. For instance, as a researcher mentioned

I believe that my organism, my immune system is able to fight against seasonal flu. […] So honestly, I prefer to maintain my defenses, my lymphocytes in combat mode instead of telling them: ‘Ok don’t move, the treatment takes care of business!’ | QNSEF04

Last, although this consideration would appear at first glance to be less of a priority, some interviewees based their acceptability judgment of nanocarriers on a consideration of potential effects on the health of all the workers involved in the treatment’s life cycle:

When we develop a new treatment, we must be concerned not only by patients’ health, but also by the production and the evacuation circuit of the drug. […] There are, in addition, some concerns related to the hospital staff’s health. Those health-related concerns should also be part of the evaluation before the commercialization of any new pharmaceutical molecule. | QSSHF04

#### Environment

In regards to the environment, interviewees mentioned impacts related to pollution and sustainable development. Pollution caused by the release of residues during the production of nanocarriers was one negative consequence perceived as likely and often invoked to account for a part of an interviewee’s reluctance to use the nanocarriers. Secondary pollution caused by treated patients’ elimination of the metabolized nanocarriers was another facet of environmental pollution that some respondents considered, to a smaller scale than pollution created during the production process. In the researchers for whom the sustainable development of new medical technologies is a significant concern, the use of materials that are abundantly available (carbon) and potentially biodegradable (synthetic DNA) was a positive facet of the nanocarriers.

#### Social Cohabitation

The issue of social cohabitation is the one with the broadest range of impacts perceived and invoked in explaining acceptability judgments about the two kinds of nanocarriers. It includes questions of access and inequality, social burdens associated with treatment, societal consequences of disease, freedom to choose the treatment offered, and productivity of sick people. The point was made that the development and use of such treatments, assuming they prove efficacious, could mitigate the consequences that certain diseases have for society. Specifically, the use of these treatments could decrease the slowdown that occurs in certain areas of operation during the flu season and reduce the amount of resources assigned to treating lung cancer patients. As well, for some of the researchers interviewed, the development of these nanocarrier-based treatments was deemed acceptable, not based on these respondents’ own intention to use them, but because they considered freedom of choice in treatment important:

Each person should have the choice. I made my choice for certain reasons and someone else could have very good reasons for making a completely different choice than mine. We should give everybody the possibility to make their own choices. I can understand that it’s not everyone who has the ability to analyze all this information, but if we let science go forward and it’s properly overseen, then we’ll advance cautiously. Each person can make their own choice and my choice is certainly not the best one for another person. | QSSHC02

On the other hand, some of the researchers interviewed believed that consideration should also be given to the social burden, in the form of both monetary cost and changes to current practice, represented by the integration of these new treatments into the health system. Access and inequality as possible barriers to the acceptability of these treatments were deemed relevant, where the considerations mentioned related to patients in developing countries having limited access to these new technologies and to potential cleavages between social classes within the same country. As well, a concern about one’s occupation and the impact of his or her illness on the society was the object of reflection on the acceptability of a highly effective treatment. On one hand, such a treatment could put some individuals with important roles in society whose unforeseen prolonged sick leave could impact many people—the example given was that of a surgeon—back on their feet rapidly. But, in contrast to this, some researchers spoke of their fear that such an efficacious treatment could result in social pressure on patients, driving them to resume work activities to soon, to the detriment of the process of full recovery.

#### Economy

Impacts perceived and weighed in connection with the economy clustered around three main factors: the cost of developing the treatment, the cost at the time of treatment, and economic considerations related to marketing. Several of the researchers interviewed raised the possibility that treatments of such complexity could be costly to develop in comparison with other choices. A high development cost could mean higher costs at the time of treatment, with possible consequences for the treatment’s market cost and its recognition by insurance companies. This perspective led to acceptability judgments against the TDD. In contrast, some other interviewees thought a technology of this kind might be less costly to develop, a consideration that favored its acceptability. The individual cost to the patient at the moment of purchase was a concern for respondents, and the significance assigned to financial considerations in relation to a treatment’s acceptability appears to be in inverse proportion to the seriousness of the disease. The potential for economic benefits is another facet of the economic impact in its relation to acceptability judgment. These benefits could be small scale, applying only to a pharmaceutical company, or large scale, with an impact on a nation’s health care system. For example, a country may develop the treatment, may become the international standard for it, and thus would not need to buy the treatment abroad. Some researchers who were concerned with these impact facets valued them positively within their acceptability judgments.

#### Life and Death

The issue of life and death was also brought up by some respondents in expressing their acceptability judgments about the treatments presented in the scenarios. On one hand, there was the possibility that a TDD treatment would result in improved health, thus in life expectancy, for example, for someone with a serious case of cancer. Going further still, some of the researchers placed emphasis on the possibility that such a treatment could cure a disease with a history of high mortality. On the other hand, the perception that a treatment might have adverse effects whether in the short or the long term, resulting in death, seemed to negatively impact the acceptability of the new treatments.

#### Representations of the Human Being and Nature

The effect of a nanocarrier-based treatment on human bodily identity, on the integrity of the physical self, and on the definitions of health and sickness are all impact facets that were considered by the researchers interviewed. The alien nature and “artificial” quality of nanocarriers gave rise to reflection on the acceptability of the TDD treatments presented. While recovery of an optimal state of health was a change deemed to be a desirable effect of using these treatments, the physiological consequences of genetic alterations caused by nanocarriers negatively impacted the acceptability of nanocarriers. These potentially undesirable changes that the nanocarriers might produce in the human body were deemed by some of the researchers to weigh against the treatment’s acceptability, in light of the importance of the integrity of the human body. Last, the point was made that a way of practicing medicine as efficacious as this and one that targets nanoscale mechanisms could potentially redefine the nature of health and sickness and lead to a paradigm shift in the field of medicine.

#### Technoscience

Two opposing impacts related to the issue of technoscience were invoked in support of acceptability judgments of respondents concerning the nanocarriers presented in the scenarios. Several people considered the advancement of science and the development of these medical treatments to be an important engine of scientific progress. Some of the researchers interviewed also raised the importance of technoscientific advances in medicine as a springboard for the solution of certain significant technological locks. But, while some interviewees embraced this perspective of the technological springboard, others simply challenged the scientific relevance and the appeal of an undertaking like the development of a nanocarrier to treat the seasonal flu, as may be seen in this excerpt from an interview:

Developing a research program as expensive as I know them to be for this type of technology, just to treat the flu, I wonder if maybe the treatments we already nowadays have aren’t enough. | QSSHF01

### The Influence of Contextual Factors on the Acceptability Judgment

In the wake of the first analysis, it was observed that certain contextual factors might influence the way the impacts and impact facets presented above were weighed and placed in relation to each other within the acceptability judgments of the researchers interviewed. Accordingly, a second analysis of the interview materials was undertaken to identify these contextual factors. The interview coding frame was initially based on the theoretical framework operationalized in the study but did not allow for the researcher’s judgment to be resituated in its context. An approach by emerging codes consistent with the general inductive analysis approach allowed for the identification of different factors that were related to the context and that had been coded at first under the categories *alternatives*, *efficacy*, *uncertainties*, *use*, *user*, and *usefulness*. The meaning units that had emerged from that initial coding work were analyzed and organized hierarchically, with the result that three major categories, *device*, *use*, and *user*, of contextual factors influencing researchers’ acceptability judgments surfaced and served to organize subcategories. On this basis, a second iteration in the coding process was conducted and resulted in the emergence of all the subcategories of each major theme. Finally, a more in-depth analysis of the recoded meaning units allowed for fine-tuning the facets of all the subcategories related to contextual factors. Table 3 presents all those contextual factors modulating the acceptability judgment.

**Table 3:** Device, use, and user as contextual factors modulating the acceptability judgment about TDD nanocarriers presented in the scenarios

Device	Nature of the device	*Carbon*
		*Synthetic DNA*
Use	Gravity of the disease	*Benign*
		*Fatal*
	Purpose of the treatment	*Cure*
		*Comfort*
	Efficacy of the treatment	*Clinically proven*
		*In comparison with another treatment*
	Alternatives to the treatment	*Possible prevention*
		*Existing other treatment*
		*Non-pharmacological alternative*
	Areas of uncertainty	*Treatment’s stage of development*
		*Progress of the disease*
User	State of health	*Initial state of health*
		*Stage in the disease’s progress*
	Psychosocial features	*Perceived usefulness*
		*Sociodemographic characteristics*
	Social interactions	*Trust in the physician*
		*Influence of loved ones*

#### Contextual Factors Related to the Device

Two nanocarriers for TDD have been presented to respondents (carbon, synthetic DNA). However, it was stated that the active pharmacological ingredient inside both nanocarriers was the same, depending on the clinical context of use. Consequently, in the context of this study, most of the interviewees have not attached importance to the nature of the devices, as presented in those two excerpts:

Has the treatment been tested? Is it mature? Because after all, nano or not, carbon or not, I don’t really care. If the results are there, they are there. | QSHSF01

Well, it changes nothing for me, to have a Volkswagen or a small Ford. You know, what really matters is to reach the destination | QSSHC03

#### Contextual Factors Related to Use

The gravity of the disease, the treatment’s purpose, its efficacy, alternatives to the treatment, and areas of uncertainty were the headings under which use-related contextual factors contributed to the acceptability judgment about the TDD nanocarriers were classified. The gravity of the disease is a factor that greatly influenced the balance between positive and negative impacts that entered into the acceptability judgment. For example, it was observed that in the case of a grave and potentially fatal disease (lung cancer), greater risks to health, the environment, and society were deemed acceptable in the expectation of benefiting from such positive impacts as improved state of health and indeed the possibility of survival:

In this case, my decision is reinforced by the severity of the disease. What I mean is that considering the extent to which it is difficult to treat lung cancer, I would say that if the treatment can help healing me, I would take it. | QSSHF03

The case of a more benign disease, such as the seasonal flu, made respondents less inclined to accept such high risks, as this researcher presents:

In my case, the seasonal flu doesn’t put my life in danger. So I’m not willing to accept the risk of taking something I don’t know the long term side effects, even though they are minor, in the short term. | QSSHC05

The purpose of the treatment appeared similarly to influence the way negative and positive impacts were weighed in relation to a treatment’s acceptability. In the case of a treatment aimed at curing a pathological condition, some undesirable effects on health and the environment appeared to be tolerated. However, questions about other issues, such as technoscience or representations of the human being and nature, appeared to take second place in forming the acceptability judgment, in contrast to treatments aimed mainly at the patient’s comfort.

A treatment’s efficacy can also affect its acceptability. Positive impacts for health appear to be more heavily emphasized when a treatment’s efficacy has been clinically proven. Moreover, if a treatment’s efficacy has been shown to be greater than the efficacy of alternative options and since it is more effective in promoting health recovery and indeed prolonging or saving life—considerations given a very high priority in the health context—that treatment will be preferred. This last point provides a good transition to the subject of the influence of existing alternatives to the proposed treatment. In the absence of therapeutic alternatives for a given pathological condition, the weighting assigned to the impacts on prioritized issues will be such as to make greater risks to health, the environment, the economy, and social cohabitation acceptable. Yet if therapeutic alternatives are available, the acceptability judgment will be based on the optimal balance of all prioritized facets. In a clinical context, these facets often include health, life and death, the environment, the economy, and social cohabitation. When it comes to social acceptability, prevention emerges as a possible alternative to recourse to new treatments since the cost of developing such new treatments seems disproportionate when compared with what is needed for an effective prevention campaign. The degree of uncertainty at the time of forming an acceptability judgment about a treatment may also influence what factors are taken into account and prioritized in the decision. Several of the researchers interviewed expressed the fear that using a treatment still under development could result in more severe adverse effects, which would offset the possible positive health effects. Uncertainty about the progress of the disease may also lead to a reconsideration of expected positive impacts. For a well-monitored case of cancer, whose progress is under control, a conventional treatment with known adverse effects and probable moderate positive impacts on health might be preferred. On the other hand, a case of cancer whose progress is erratic and sometimes severe might allow more for the consideration of a treatment with a somewhat less favorable overall impact on health.

#### Contextual Factors Related to the User

State of health, psychosocial features, and social interactions are contextual factors specific to the treatment’s user, which also influence acceptability judgment. The initial state of health of the person to be treated may play a significant role in her or his acceptability judgment, depending on how major the consequences will be of being treated or left untreated. For a vulnerable elderly population, the undesirable effects and health complications ensuing with a lack of treatment for seasonal flu may be conducive to the assignment of less value to the undesirable effects of treatments such as those presented in this study. For an individual in very good health, for whom recovery from the flu without therapy of any kind presents no special risks, the assessment of the treatments presented in this study could be very different. The stage reached by the disease may also affect an acceptability judgment about a treatment. For example, in a case of lung cancer, an acceptability judgment about the offer of a first-line treatment might involve the balancing of totally different issues than an acceptability judgment in response to the offer of a third-line treatment. At the start of cancer treatment, survival and the disease’s remission are positive impacts likely to be more heavily prioritized in a decision about recourse to a treatment. In the terminal stage, the desire for acceptable quality of life, even if at the cost of life expectancy, could tip the scales in the other direction if a treatment’s undesirable effects appear significant.

In connection with the contextual factors that could modulate the acceptability judgment about the treatments, perceived usefulness and sociodemographic characteristics are two facets that fall under the umbrella of psychosocial factors. On one hand, the perceived usefulness of the treatments presented appeared to have played a major role in their acceptability. In the contexts presented, perceived usefulness appears to have been defined in relation to the desired positive impacts of a treatment on prioritized issues (e.g., improved quality of life, prolonged life expectancy, and lower medical costs). A perception that the proposed treatment would not lead to the achievement of these hoped-for positive impacts—that is, a perception that it would be useless—impedes reflection about individual acceptability, making it impossible for the person to weigh other perceived impacts. On the other hand, sociodemographic characteristics appeared to function as significant modulating factors in the acceptability judgment. Respondents’ cultural profile (French, Canadian), their disciplinary profile (NSE, SSH), their current life circumstances, and their ages were all factors that colored their decisions to use or not to use the treatments presented. For example, although all the researchers invoked both positive and negative impacts, an initial finding about cultural differences shows that the French researchers were more likely to invoke negative impacts in forming their acceptability judgments about carbon nanocarrier use to treat the seasonal flu. The French researchers mainly identified adverse effects and problems of unequal access to treatment, environmental pollution, and high production costs as the basis for their disagreement with the treatment. In contrast, despite their low rate of acceptance for the treatment in question, the Canadian researchers emphasized the treatment’s potential positive impacts. Particular attention was given to the desirable health effects for the individual receiving the treatment and the management of a disease that is potentially fatal for some vulnerable populations. Other positive impacts relating more to the general context of care were also taken into account in reaching an acceptability judgement. In regards to the nature of the issues discussed, the French researchers had a more pronounced tendency to bring up themes related to the economy, the environment, and technoscience, mentioning the high cost of development of such a specialized technology and environmental pollution and challenging the idea of this type of treatment. These researchers also raised some concerns about the inequality which may arise as to the use of this treatment. The Canadian researchers for their part emphasized issues of social cohabitation in a different manner, stressing the importance of being able to choose whether or not to be treated this way—thus endorsing the importance of a choice being possible for all members of society even if they themselves did not agree with personal use of the treatment—and of the productivity of the individuals treated. Results related to disciplinary distinctions were reported in another publication dedicated to that objective (see [21]). Respondents’ age at time of treatment also seemed to influence the weight they assigned to certain impacts as related to time of life. Long-term undesirable health effects (i.e., appearing for at least 20 years following exposure) were perceived as less concerning for a retired person than for a young person. Thus, it was more acceptable to take a new treatment when it appears that such long-term effects are unlikely to develop.

Finally, social interactions such as those between patient and physician and the influence of loved ones were also deemed likely to influence a treatment’s acceptability in clinical context. A physician’s assent to a treatment’s use and the bonds of trust between patient and attending physician sometimes appear to make the exercise of exploring a patient’s acceptability judgment pointless, since the physician’s judgment supplants the patient’s. Trust in an expert’s opinion in a given situation could be so heavily emphasized that, regardless of the patient’s acceptability judgment, the physician’s prevails. Along with the patient–physician bond of trust, the influence of the family and the role played by the treated individual within her or his circle are the final contextual factors that may have influenced acceptability judgments of the treatments presented. On one hand, a spouse’s opinion may influence the weight one assigns to negative and positive impacts in arriving at an acceptability judgment. On the other hand, the possibility that the disease will have consequences for loved ones (children, partner, parents) may provide a context in which the acceptability judgment is arrived at via a less self-centered weighting of possible impacts.

## Discussion

This study’s first objective was to present the full range of the issues taken into account and weighed when arriving at acceptability judgments concerning the use of TDD nanocarriers in the two clinical contexts presented. A description of this wide spectrum intended to reveal how rich and diverse the full range of perceived and weighed impacts is when formulating acceptability judgments. Also, this exercise aimed to highlight how both ELS implication and EHS issues are intertwined and simultaneously characteristic of nanotechnological applications under development. This range was presented in relation to the impacts and impact facets invoked by the researchers interviewed in explaining their intention to each of the two treatments in each scenario presented, as well as in relation to the weighting respondents assigned to these impacts in arriving at their acceptability judgments. As for the study’s second objective, this descriptive analysis also allowed for a more detailed exploration of the contextual factors underlying the variability of certain acceptability judgments in a clinical context. This helped strengthen the fact that the acceptability analysis of any new technology has to be contextualizing regarding three main elements that are the device, the user, and the context of use.

In regards to the first objective, it was found that the researchers who formed acceptability judgments about these projected TDD nanocarriers in the specified clinical contexts based their judgments on a wide spectrum of positive and negative impacts. In line with the theoretical framework chosen for the study [7], each of these impacts was classified under one of seven issues: health, environment, social cohabitation, economy, life and death, representations of the human being and nature, and technoscience. This first finding supports the view of those authors who recommend bringing an integrative and qualitative approach to the assessment of the acceptability of new technologies such as NM applications. By incorporating the diverse E^3^LS concerns of researchers, potential user, and even general public (all stakeholders who are likely to raise considerations beyond those of safety and toxicity) and by elaborating the precise nature of potential impacts perceived and weighed in acceptability judgment [3, 5, 16, 30], the status of emerging technologies like NM applications could be switched from marketable products, to value-laden technoscientific objects. This new perspective toward emerging technologies could encourage by the same way responsible innovation.

Concerning the prioritization of issues, the impacts of aspects related to health, life and death, environment, social cohabitation, and economy weighed more heavily in the researchers’ formation of their acceptability judgments. This assessment reveals certain priorities based in the user’s perspective that play a role in clinical decisions. On the other hand, the study’s findings also support the position of Senjen and Hansen [16], since the researchers interviewed also perceived and considered impacts on certain more metaphysical issues in arriving at their acceptability judgments. The potential for the modification of the human body by nanocarrier action was considered by some researchers, in some cases positively and in some cases negatively. This potential was considered in relation to the significance they assigned to the representation of the human being and nature as a value in itself. The possible impact of the advent of a new, nanoscale, era in medicine and the consequences of this on definitions of health and sickness, not to mention of science and technology, were also considered in relation to newly developed nanomedical applications. These questions appeared to be assigned a lower priority in the context of a clinical decision. A hypothesis may be formulated suggesting that those impacts and related concerns are less important for the user in a medical context but may be more important to consider upstream, during the process of technological development. This hypothesis supports the perspective taken by Boenink [30] in discussing ethical issues associated with the development of molecular medicine. It also highlights the possibility that concerns and perceived and weighed impacts evolve over time and depend on the context, as discussed in a next section.

The methodological approach taken in this study corrected for the weaknesses of perception studies identified by Patra et al. [31], who underlined the lack of perception of ethical issues by the NT practitioner-researchers who were interviewed as a limitations in their own study. The traditional modes of operationalization in approaches to impact perception and to acceptance often bring out no more than the main issues, with no greater examination or fine-tuning of potential impacts for specific issues. While it is clear that health is the issue assigned the highest priority in the clinical contexts chosen, this study has shown that it is possible for multiple impacts on health to exist and for these impacts in turn to present numerous facets, each of which is assigned a weight independently of the others. Impact on health can be viewed as a series of distinct kinds of impact, such as desirable effects or well-being. Under desirable effects, recovery from the disease or mitigated symptoms is often considered. This range of impacts on prioritized issues creates a much broader portrait of acceptability judgments about a given application. From the perspective of responsible development and of a participatory approach, drawing up a list of the impacts perceived and prioritized by researchers involved in the reflexive and developmental aspects of an emerging technology, which are also developing technology’s potential users, could make it possible to intervene very early in the innovation process. Thus, such an approach integrating E^3^LS issues and centered on acceptability may help to pave the way for the design and implementation of more valuable applications ([32]; quoted in [33]) or treatments, in the case of NM.

In regards to the second objective, it was found that certain contextual factors underlay the variability in acceptability judgments about TDD nanocarriers. On the nature of the device, the inductive analysis revealed that, for the scenarios presented, most of the interviewees have not attached importance to whether the nanocarrier was made of carbon or synthetic DNA. The initial choice of those two envisioned nanocarriers, inspired by analyses of current state of the art on nanocarrier-based TDD, was intended to reveal the potential effect of the nature of the treatment on acceptability judgments of respondents, yet proved to be unimportant in the balance. Different methods of administration of the treatments presented (i.e., an oral nanocarrier-based TDD treatment vs an intravenous nanocarrier-based TDD treatment) may have more of an impact on how acceptability judgments were formulated. Nonetheless, some interviewees mentioned different impacts for each nanocarrier presented but stated that their knowledge was not acute enough regarding NM to pretend that those differences had a real impact on their acceptability judgment. Moreover, the relatively low effect of the device itself on acceptability judgment supports taking a step backward and integrating other contextual factors (use and user) that are required to conduct an enlightened analysis of acceptability.

With respect to use, whereas the nature of the nanocarrier has been shown to have no effect on acceptability judgments about the proposed treatments [20], it was found that the seriousness of the disease and the purpose of the treatment do influence the weighting assigned to perceived impacts. These findings agree with those of Gupta [23] and Satterfield [9], who observed the influence of, respectively, the application and the context on perceived impacts, social acceptance, and respondents’ acceptability judgments. This also confirms what was reported by Severin et al. [34], who found that the purpose of a genetic screening test could influence its acceptability to clinical geneticists and other stakeholders. In addition to the nature and the purpose, a treatment’s efficacy is also a documented contextual variable traditionally taken into account in the development of approaches based on technology acceptance models [35, 36]. The emergence of this variable in the clinical contexts presented could be linked to the preponderant weighting assigned to positive health impacts in arriving at an acceptability judgment about a treatment. A treatment’s efficacy, or its capacity to restore the patient to an optimal state of health, is likely to influence this judgment. However, it is possible that the gap in the magnitude of the relative severity of each context of use may have polarized responses. More similar settings may have generated less variation in researchers’ acceptability judgments. The existence of treatment alternatives also emerged as capable of modulating the acceptability judgment. The work of Fisher [37] also incorporated this insight through its acknowledgment that the existence of alternatives can influence how weight is assigned to the prioritized factors in a given situation. Finally, the presence of elements of uncertainty was also noted as potentially influencing acceptability judgments. The theoretical framework that underlies this study incorporates the dimension of uncertainty in specifying that a perceived impact’s probability of occurrence is not always known [7]. Contextual uncertainty is presented here from a new perspective, not as an unknown, but as a condition on which different outcomes could result in varying ways of weighting the impacts perceived in a given situation.

User characteristics that were revealed to function as modulating factors in the acceptability judgment were general state of health, psychosocial features, and social interactions. The literature on impact perception and acceptance supports findings on the influence of psychosocial features. Perceived usefulness [35, 36] and potential users’ sociodemographic characteristics [9, 19, 34], in particular, social culture as manifested in values and reference points [38–41] and disciplinary culture [21], are contextual factors that emerged during the interviews and that align with current practices in technology assessment studies. Given the range of the possible E^3^LS issues regarding NT applications and regarding cultural sensitivities throughout France and Canada for instance, new approaches will have to bridge the gap between researchers’ discourse in an interdisciplinary and an international manner. Social interactions, viewed in the behavioral psychology literature as social norms [42], are also generally believed to influence an individual’s intention to use a technology [35], as well as having been identified by researchers as a source of the variability in value judgments relating to intention to use a medical treatment. Lastly, the present findings about the user’s general state of health are consonant with previous findings that acceptability judgments about intention to use a treatment vary according to the vulnerability of the individual or population to be treated [21]. The use of this last criterion could be extended beyond the study of the individual and social acceptability of a treatment’s use to the earliest stages of development of new medical treatments.

Rooted in the respondents’ perspective, one major strength of this study is the place assigned to the voice of the researchers in the process of constructing the range of impacts that could result from the use of the nanocarrier-based TDD treatments presented. Traditional approaches to impact perception and acceptability have focused on prediction and generally target the determinants of impact perception and acceptance. In contrast, here the voice of the researchers has helped to better understand their reflections and situate them within the patient/user’s perspective, resulting in the emergence of a general portrait of nanocarriers for TDD. Moreover, the range of perceived impacts and impact facets featured in the study constitutes an innovative portrait of nanocarrier for TDD in relation to the specified clinical contexts of use. That portrait could serve as a model available to stakeholders who wish examine the acceptability of TDD nanocarriers in-depth. It could promote their reflectiveness with respect to the diverse possible weightings that the potential users of NM might assign to perceived impacts. The perspectives emerging from this study could also serve as a reference for future undertakings for the study of other NM applications or of NT in general. Further, the contextual framework connecting the device, the use, and the user is not unique to NM or NT. Consequently, in order to structure the fundamental elements prior the conduction of any analysis of acceptability, it could be applied to other technological contexts. However, this study’s design did not make it possible to take into account the temporality of the acceptability judgment as a potential modulating factor [43]. The scenarios evoked the projected use of envisioned applications, and a first hypothesis might be that the acceptability judgment varies in time because concrete contact with a real application could influence impact perception and weighting. A second hypothesis related to temporality could be that the same applications might be assessed differently by researchers if, someday, they are really facing the described clinical situations.

## Conclusion

Interviews with researchers in the field of emerging technologies allowed for the creation of a portrait of perceived impacts and how those impacts were weighed in response to two envisioned applications for nanocarrier-based TDD in two clinical contexts of use. It was concluded that impacts were perceived and weighed from the perspective of a set of considerations combining ELS implications and EHS issues, namely, ethical, environmental, economic, legal, and social (E^3^LS) aspects. It was also found that the set of impacts on issues could be fine-tuned to reveal diverse facets that are important to take into account in order to flesh out potential users’ acceptability judgments and give them greater precision. This approach, focused on the perceived impacts and their weighting within the process of arriving at an acceptability judgment, encourages going beyond traditional approaches aimed at predicting perceptions and technological acceptance. The E^3^LS approach presented could contribute to a united effort in the development of new methodologies allowing for an understanding of the user’s perspective, an approach that is consonant with the need for social dialogue on the question of the development of new technologies and deemed feasible in the context of the development of new medical technologies [44]. It was also concluded that, in the effort to make technological development participatory, it is necessary to contextualize acceptability judgments of potential users, taking note of who is passing judgment, and to take account of contextual factors that foster variability in acceptability judgments about a new application. Such a consideration of users is behind steps that have already been taken to change methods of governance and the composition of decision-making bodies. This aspect is visible in the health field, where we see the inclusion of users and clients on assessment committees, and in the emergence of user-centered advisory committees. Giving effective consideration to these contextual factors could add a supplementary layer of information to assessment procedures, so that judgments by all stakeholders may come together and produce a better picture of the ethical acceptability of NM, opening by the same way a dialogic space on technological innovation.
